# MicroRNA-125b suppresses the migration and invasion of hepatocellular carcinoma cells by targeting transcriptional coactivator with PDZ-binding motif

**DOI:** 10.3892/ol.2015.2973

**Published:** 2015-02-17

**Authors:** JIPENG LI, LAIFU FANG, WANJUN YU, YIPING WANG

**Affiliations:** 1 Department of Clinical Laboratory, Yinzhou People’s Hospital, Ningbo, Zhejiang 315040, P.R. China; 2 Department of Pathology, Yinzhou People’s Hospital, Ningbo, Zhejiang 315040, P.R. China; 3 Department of Respiratory and Critical Care Medicine, Yinzhou People’s Hospital, Ningbo, Zhejiang 315040, P.R. China

**Keywords:** microRNA-125b, migration, invasion, hepatocellular carcinoma, transcriptional coactivator with PDZ-binding motif

## Abstract

MicroRNAs (miRNAs) are a class of small non-coding RNA molecules that serve an important function in carcinogenesis and tumor progression. The present study investigated the roles and mechanisms of miRNA-125b (miR-125b) in human hepatocellular carcinoma (HCC). miR-125b was significantly downregulated in the examined HCC tissues and cell lines. Overexpression of miR-125b reduced HCC cell migration and invasion. By contrast, inhibition of miR-125b expression significantly accelerated HCC cell migration and invasion. In addition, the present study identified transcriptional coactivator with PDZ-binding motif (TAZ) as a functional downstream target of miR-125b. Furthermore, overexpression of TAZ impaired miR-125b-induced inhibition of invasion in HCC cells. The current study demonstrated that miR-125b may be involved in the tumorigenesis of HCC at least in part by the suppression of TAZ.

## Introduction

Hepatocellular carcinoma (HCC) is the sixth most prevalent type of cancer worldwide and the fourth most common cause of cancer-associated mortality (1,2). Currently, surgical resection and transplantation are the most effective treatment approaches for HCC (3). However, the recurrence rate within 2 years in patients who have undergone tumor resection remains >50% (4,5). Uncontrolled tumor metastasis, frequent intrahepatic spread and extrahepatic metastasis are the primary causes for the poor prognosis in HCC (6). Therefore, improved understanding of the molecular mechanisms that underlie HCC invasion and metastasis is essential for the development of novel therapeutic strategies.

MicroRNAs (miRNAs) are a class of small non-coding RNA molecules that negatively regulate the expression of target genes by mRNA degradation or translational inhibition. Previous evidence has indicated that the dysregulation of miRNAs may lead to alterations in diverse biological processes, including proliferation, differentiation and apoptosis, which are associated with the development of cancer (7,8). Several dysregulated miRNAs, including miR-221, miR-21, miR-452, miR-424 and miR-125b, have been demonstrated to regulate HCC cell growth, apoptosis, migration and/or invasion (9–13). However, the role and the underlying molecular mechanisms of miR-125b in HCC remain largely unknown.

To investigate the possible role of miR-125b in HCC, the current study investigated miR-125b expression levels in HCC tissue relative to surrounding healthy tissue. In addition, the migratory and invasive properties of HCC cells were investigated in the presence and absence of miR-125b expression.

## Materials and methods

### Tissue samples, cell lines and cell transfection

Specimens from HCC and surrounding control tissue were obtained from 20 patients at Yinzhou People’s Hospital (Ningbo, China) prior to definitive therapy. The tumor tissues and adjacent normal tissues were frozen in liquid nitrogen following resection. Informed consent was obtained from all subjects, and the study was approved by the review board of the hospital ethics committee.

Four HCC cell lines (SK-Hep-1, SMMC7721, HepG2 and Huh7) and a normal liver cell line (L02) were purchased from American Type Culture Collection (ATCC) and cultured in Dulbecco’s modified Eagle’s medium (Gibco Life Technologies, Carlsbad, CA, USA) containing 10% fetal bovine serum (FBS) with 100 U/ml penicillin and 100 μg/ml streptomycin at 37°C with 5% CO_2_.

miR-125b mimics, negative control mimics (NC), miR-125b inhibitor (anti-miR-125b), negative control inhibitor (anti-NC) and TAZ short-interfering RNAs (siRNAs) were synthesized by Shanghai GenePharma Co, Ltd., (Shanghai, China). Transfection was performed with Lipofectamine 2000 (Invitrogen Life Technologies, Carlsbad, CA, USA) according to the manufacturer’s instructions. In brief, for each well, 5 μl mimics, inhibitor or siRNA (20 μM) was added into 250 μl Opti-MEM medium (Gibco Life Technologies, Carlsbad, CA, USA), 5 μl of Lipofectamine 2000 into 250 μl Opti-MEM medium, and then mixed mimics, inhibitor or siRNA with Lipofectamine 2000. The mixture was added to cells and incubated for 6 h before replacing the medium. Total RNA and protein were prepared as described below 48 h subsequent to transfection and were used for reverse transcription-quantitative polymerase chain reaction (RT-qPCR) or western blot analysis.

### Plasmid construction and luciferase reporter assay

This was performed as described in our previous study (14). In brief, the wild-type 3′-untranslated (3′UTR) region of TAZ, containing predicted miR-125b target sites, was amplified by PCR from SK-Hep-1 cell genomic DNA. The primer sequences were as follows: F 5′-GAT CTG CAG CTC TCC CAG GGG CTG GCT TCA G-3′ and R 5′-GAT CAT ATG GAG GCA GAA AGG ATG GAG AAG T-3′. The corresponding mutant constructs were created using the QuikChange site-directed mutagenesis kit (Agilent Technologies, Inc., Santa Clara, CA, USA).

The wild-type and mutant 3′UTR fragments were subcloned into the pGL3-control vector (Promega Corporation, Madison, WI, USA) immediately downstream of the stop codon of the luciferase gene. The DNA fragment encoding the TAZ protein was amplified by PCR from SK-Hep-1 cell cDNA, and cloned into a pCMV-Myc expression vector (Clontech Laboratories, Mountain View, CA, USA). The primer sequences were as follows: F 5′-GCT GAA TTC GAC CTA GAG GCG CCC CAC AGG C-3′ and R 5′-CTG CTC GAG TCT GTG CGG GCC AAG AAT CCA G-3′. For luciferase assays, the reporter plasmid was co-transfected with a control *Renilla* luciferase vector (Promega Corporation) into SK-Hep-1 cells in the presence of either miR-125b or NC. After 48 h, cells were harvested and the luciferase activity was measured using the Dual-Luciferase Reporter Assay System (Promega Corporation).

### RNA extraction and RT-qPCR

RNA extraction and RT-qPCR was performed as described in our previous study (14). In brief, total RNA was extracted from the cultured cells and the HCC tissue specimens using TRIzol reagent (Invitrogen Life Technologies) according to the manufacturer’s instructions. The expression level of mature miR-125b was measured by TaqMan miRNA assays (Applied Biosystems Life Technologies, Foster City, CA, USA) according to manufacturer’s instructions and normalized against U6 small nuclear RNA levels. TAZ expression was measured by SYBR green qPCR assay (Takara Biotechnology Co., Ltd., Dalian, China) and GAPDH was used as the endogenous control.

### Western blot analysis

Western blotting was performed as described in our previous study (14). In brief, protein extracts from cells were prepared using a modified RIPA buffer with 0.5% sodium dodecyl sulfate (SDS) in the presence of Complete Mini protease inhibitor cocktail (Roche Diagnostics GmbH, Mannheim, Germany). Polyacrylamide gel electrophoresis in 10% SDS gels with low voltage (60 V) for separating gel; use higher voltage (140 V) for stacking gel., tank-based transfer to Immobilon Hybond-C membranes (GE Healthcare Bio-Sciences, Pittsburgh, PA, USA) and immunodetection were performed with standard techniques. Antibodies were used in western blot analysis in accordance with the manufacturer’s instructions. In brief, the membrane was incubated with mouse anti-human TAZ monoclonal antibody (catalog no. H00006901-M12; Novus Biologicals, Littleton, CO, USA) and mouse anti-human β actin monoclonal antibody (catalog no. sc-47778, Beijing Zhongshan Biotechnology; Beijing, China) at 1:1500 dilution at 37°C for 2 h, and then with peroxidise-conjugated goat anti-mouse IgG (catalog no. ZB-2305, Beijing Zhongshan Biotechnology) at 1:2000 at room temperature for 1 h. Signals were visualized with SuperSignal West Pico Chemiluminescent substrate (Thermo Fisher Scientific, Inc., Rockford, IL, USA) by exposure to films.

### Wound healing and invasion assays

Cell migration was assessed by wound healing assays. Cells (2×10^5^ cells/well) were seeded in six-well plates and cultured to 100% confluence. Wounds were generated in the cell monolayer using a plastic pipette tip. The cells were then rinsed with phosphate-buffered saline and cultured for a further 48 h. The spread of wound closure was observed and images were captured using a confocal laser scanning microscope (Olympus; Tokyo, Japan) as described previously (15). For invasion assays, 2×10^5^ cells were added into the upper chamber of the insert (6.5 mm in diameter, 8 μm pore size; Corning Life Sciences, New York, NY, USA) pre-coated with Matrigel (ECM gel, Sigma-Aldrich, St. Louis, MO, USA). Cells were plated in medium without serum (Gibco Life Technologies), and medium containing 10% FBS in the lower chamber served as a chemoattractant. Following 24 h hours of incubation, the cells that did not invade through the pores were carefully wiped out with cotton wool, and the filters were fixed by treatment with 95% ethanol for 30 min and stained with 0.2% crystal violet solution (Beyotime; Shanghai, China) for 30 min. Invasive cells adhering to the undersurface of the filter were counted (5 fields/chamber; 0.24 mm^2^/field) using an inverted microscope as described in our previous study (14), and each experiment was repeated three times.

### Statistical analysis

Statistical analyses were performed using SPSS software, version 16.0 (SPSS, Inc., Chicago, IL, USA). Data from three independent experiments are expressed as the mean ± standard deviation. Differences were assessed by two-tailed Student’s t-test. P<0.05 was considered to indicate a statistically significant difference.

## Results

### Expression of miR-125b is reduced in HCC tissues and cell lines

**Figure 1:**
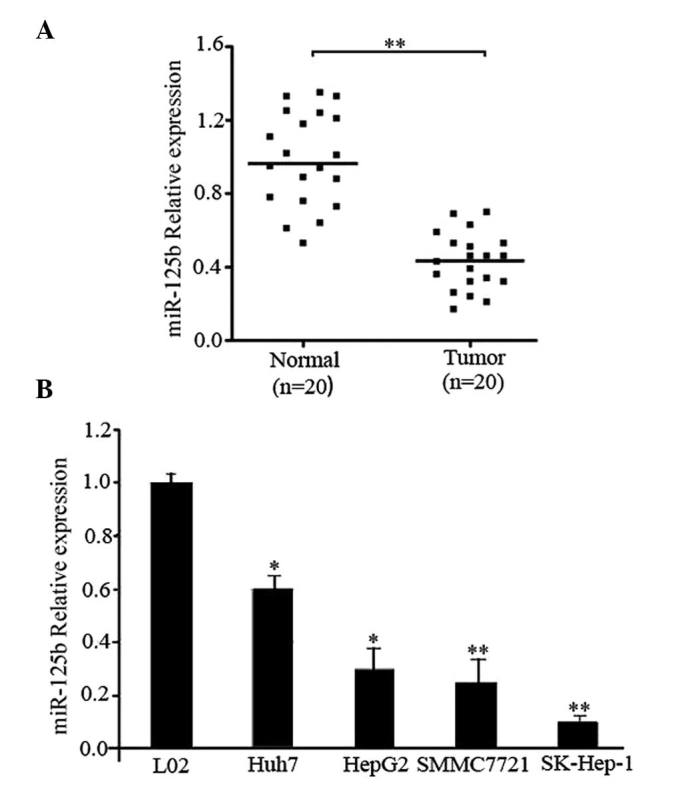
(A) Expression levels of miR-125b in 20 pairs of HCC tissues and matched normal liver tissues were measured by RT-qPCR. U6 small nuclear RNA was used as an internal control. (B) Expression levels of miR-125b in the normal liver cell line L02 and four HCC cell lines (Huh7, HepG2, SMMC7721 and SK-Hep-1). ^*^P<0.05 and ^**^P<0.01 vs. L02 cells. HCC, hepatocellular carcinoma.

In order to study the expression of miR-125b and its significance in HCC carcinogenesis, expression levels of miR-125b were measured in 20 pairs of HCC tissue samples and their corresponding control liver tissues using RT-qPCR. The results indicated that miR-125b expression was significantly reduced in HCC tissues compared with the normal tissues (P<0.01; Fig. 1A). In addition, the expression of miR-125b in the four HCC cell lines was determined. As presented in Fig. 1B, the relative expression levels of miR-125b in the four HCC cell lines were significantly reduced compared with that of the healthy liver cell line, L02 (Huh7 and HepG2, P<0.05 vs. L02 cells; SMMC7721 and SK-Hep-1, P<0.01 vs. L02 cells). These results suggest that the downregulation of miR-125b may be involved in HCC carcinogenesis.

### miR-125b suppresses HCC cell migration and invasion in vitro

**Figure 2:**
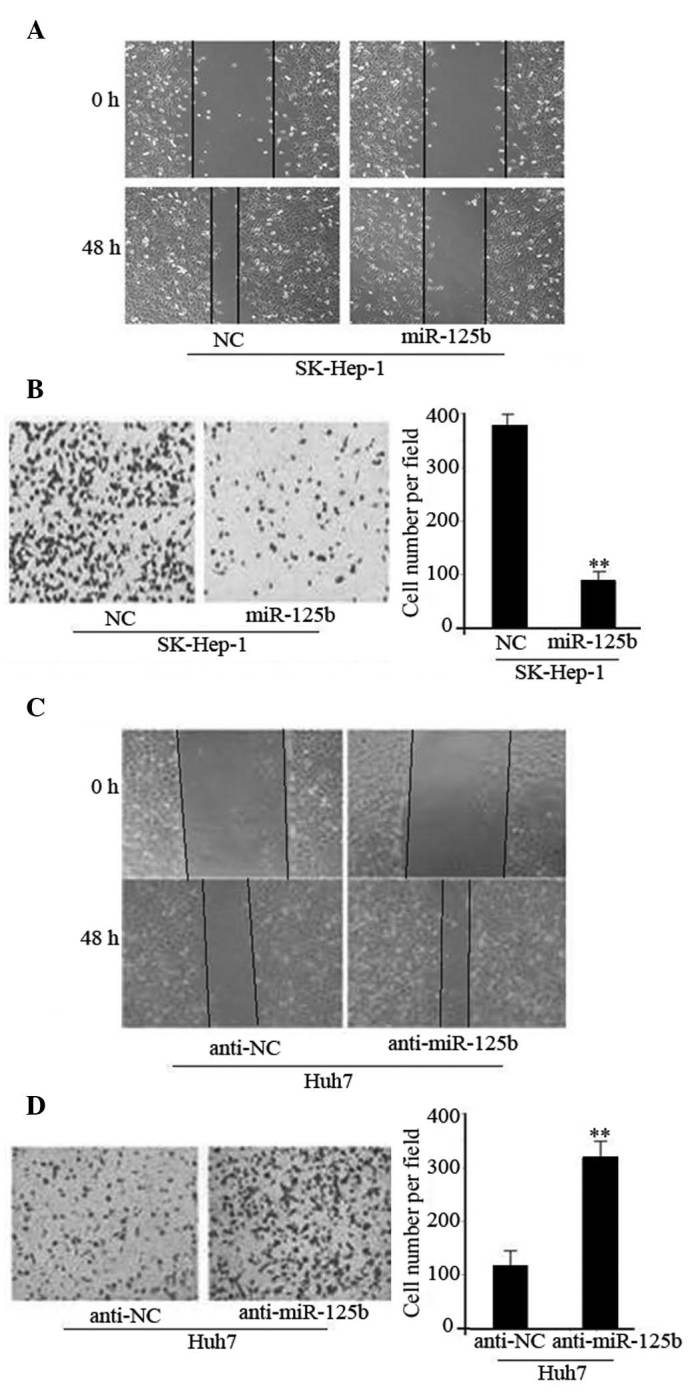
miR-125b suppresses HCC cell migration and invasion *in vitro*. Ectopic expression of miR-125b significantly impeded abilities of (A) cell migration and (B) invasion in SK-Hep-1 cells. Inversely, miR-125b inhibitor enhanced (C) cell migration and (D) invasion in Huh7 cells. ^**^P<0.01 vs. NC. HCC, hepatocellular carcinoma; NC, normal control.

In order to investigate the function of miR-125b in cell migration and invasion, miR-125b was overexpressed using miRNA mimics in the SK-Hep-1 HCC cell line; then a wound healing assay was performed. As presented in Fig. 2A, overexpression of miR-125b leads to the suppression of tumor cell mobility in the SK-Hep-1 cells compared with the corresponding controls. Furthermore, Transwell assays indicated that miR-125b significantly reduced the invasive capacity of SK-Hep-1 cells (P<0.01; Fig. 2B). By contrast, the wound healing and invasion of Huh7 cells was increased following the silencing of endogenous miR-125b using anti-miR-125b (P<0.01; Fig. 2C and D). Together, these results imply that miR-125b can suppress HCC cell migration and invasion *in vitro*.

### miR-125b downregulates TAZ by directly targeting its 3′UTR

**Figure 3:**
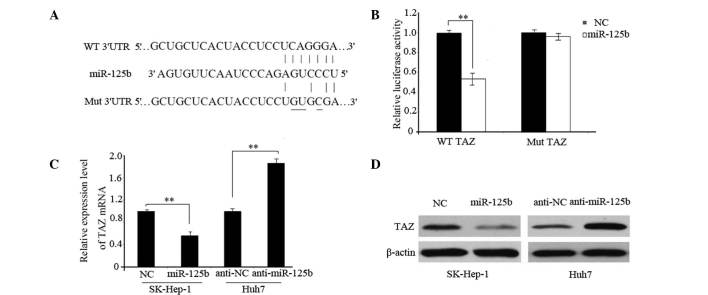
miR-125b directly targets TAZ by binding to its 3′UTR. (A) The predicted miR-125b binding site within TAZ 3′UTR and its mutated version by site mutagenesis are presented. (B) Luciferase assays indicated that miR-125b downregulated the expression of TAZ by binding with its 3′UTR. In comparison with negative controls, miR-125b inhibited TAZ (C) mRNA and (D) protein expression, while reduction of miR-125b by inhibitors moderately restored TAZ expression. ^**^P<0.01. TAZ, transcriptional coactivator with PDZ-binding motif; NC, normal control.

To investigate the molecular mechanism of miR-125b, bioinformatic algorithms (TargetScan 6.2, www.targetscan.org; and PicTar, pictar.mdc-berlin.de) were used to predict a large number of potential miR-125b target genes. Among them, TAZ was identified to possess a putative miR-125b binding site within its 3′UTR (Fig. 3A). To verify whether TAZ is the direct downstream target of miR-125b, a fragment of TAZ 3′UTR containing the putative miR-125b binding site was cloned into a luciferase reporter vector. The luciferase reporter assay indicated that the upregulation of miR-125b significantly inhibited the relative luciferase activity of TAZ 3′UTR in SK-Hep-1 cells, but did not significantly inhibit the mutant TAZ 3′UTR (Fig. 3B). In addition, RT-qPCR and western blot analysis demonstrated that the overexpression of miR-125b substantially reduced the expression of TAZ in SK-Hep-1 cells, and that knockdown of miR-125b increased TAZ expression in Huh7 cells (Fig. 3C and D). These results indicate that TAZ is a direct target of miR-125b in HCC cells.

### TAZ is involved in miR-125b-induced suppression of HCC cell invasion

**Figure 4:**
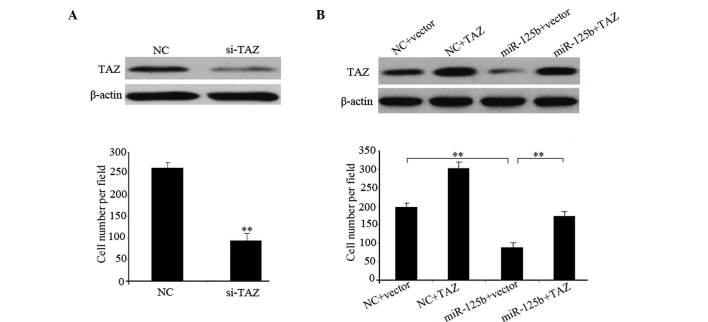
TAZ is involved in miR-125b-induced invasion inhibition in SK-Hep-1 cells. SK-Hep-1 cells were transfected with specific si-TAZ, or transfected with TAZ plasmid lacking 3′UTR along with miR-125b. Western blot analysis and Transwell invasion assays were performed to assess the effect of TAZ (A) knockdown via si-TAZ (^**^P<0.01 vs. NC) and (B) overexpression of TAZ with or without miR-125b (^**^P<0.01, comparison indicated by brackets), on cell invasion. TAZ, transcriptional coactivator with PDZ-binding motif; NC, normal control.

To determine whether TAZ acts as a critical mediator of miR-125b in HCC cells, a specific siRNA against TAZ was used to knockdown TAZ expression (siTAZ). As presented in Fig. 4A, si-TAZ significantly reduced the expression levels of TAZ protein and suppressed SK-Hep-1 cell invasion (P<0.01). To determine whether forced expression of TAZ is able to rescue the suppressive effect of miR-125b, SK-Hep-1 cells were co-transfected with miR-125b and TAZ plasmids lacking the 3′UTR region. The results indicated that forced expression of TAZ significantly rescued the inhibition of miR-125b-induced cell invasion (P<0.01; Fig. 4B). Taken together, these results indicate that miR-125b regulates HCC invasion at least in part by downregulating TAZ.

## Discussion

In the present study, the expression levels of miR-125b in HCC tissues and cell lines were measured, and the biological functions and regulatory mechanisms of miR-125b in tumorigenesis were investigated. miR-125b was downregulated in HCC tissues and cell lines and was able to inhibit cell invasion via the regulation of TAZ expression. These findings indicate that miR-125b is a notable tumor suppressor in HCC.

miR-125b is an miRNA that is expressed in neurons and astrocytes in the brain (16). The role of miR-125b in malignancies is controversial: miR-125b acts as a tumor suppressor in breast cancer, ovarian carcinoma and hepatocellular carcinoma (17–19, 13) and miR-125b expression is associated with an improved clinical outcome in liver cancer patients (20). However, in prostate cancer cells miR-125b has been demonstrated to act as an oncogene that promotes proliferation and contributes to prostate cancer pathogenesis (21). miR-125b has also been reported to negatively regulate the tumor-suppressor gene p53, and suppress p53-dependent apoptosis in zebrafish and humans (22). Consistent with previous findings in HCC (13), the functional studies presented in the current study indicated that overexpression of miR-125b significantly suppresses HCC cell migration and invasion *in vitro*.

The present study examined the molecular mechanism by which miR-125b suppresses HCC cell migration and invasion, and TAZ was identified as a direct target of miR-125b. TAZ, also termed WW domain containing transcriptional regulator 1 (WWTR1), is a WW domain-containing transcriptional coactivator that activates numerous transcriptional factors that serve important roles in the development of various tissues in mammals (23). TAZ has also been demonstrated to regulate stem cell differentiation and renewal through modulation of the transcription factors peroxisome proliferator-activated receptor-γ (PPARγ) and runt-related transcription factor 2 (Runx2), and a number of members of the SMAD gene family (24,25). In a previous study, elevated TAZ expression was observed in >20% breast cancer samples, particularly in invasive ductal carcinomas (26), which implicates TAZ in metastasis and suggests that it may increase the malignancy of breast cancer. Additionally, Zhou *et al* (27) reported that TAZ is overexpressed in non-small-cell lung carcinoma (NSCLC), and knockdown of TAZ significantly impaired the tumorigenic ability of the NSCLC cells. To the best of our knowledge, the present study is the first to demonstrate that knockdown of TAZ mimics the overexpression of miR-125b in HCC cells by suppressing invasion. Forced expression of TAZ rescued the suppressive effect of miR-125b *in vitro*, suggesting that miR-125b overexpression or siRNA-mediated downregulation of the target gene TAZ is a potential HCC therapy.

In conclusion, the present study demonstrated that miR-125b is significantly downregulated in HCC tissues and cell lines, and that forced overexpression of miR-125b in HCC cells suppressed cell invasion and migration partly through the suppression of TAZ. This finding aids the understanding of the underlying molecular mechanism of HCC carcinogenesis and provides a strong rationale to investigate whether miR-125b may act as a potential biomarker and therapeutic target for HCC in future studies.
